# Epratuzumab (humanised anti-CD22 antibody) in primary Sjögren's syndrome: an open-label phase I/II study

**DOI:** 10.1186/ar2018

**Published:** 2006-07-20

**Authors:** Serge D Steinfeld, Laure Tant, Gerd R Burmester, Nick KW Teoh, William A Wegener, David M Goldenberg, Olivier Pradier

**Affiliations:** 1 Department of Rheumatology, Erasme University Hospital, 808 Route de Lennik, Brussels 1070, Belgium; 2 Department of Rheumatology, Charite Hospital, Schumannstr 20-21, Berlin D-10098, Germany; 3 Immunomedics, Inc., Morris Plains, 300 American Road, New Jersey 07950, USA; 4 Laboratory of Hematology, Erasme University Hospital, 808 Route de Lennik, Brussels 1070, Belgium

## Abstract

This open-label, phase I/II study investigated the safety and efficacy of epratuzumab, a humanised anti-CD22 monoclonal antibody, in the treatment of patients with active primary Sjögren's syndrome (pSS). Sixteen Caucasian patients (14 females/2 males, 33–72 years) were to receive 4 infusions of 360 mg/m^2 ^epratuzumab once every 2 weeks, with 6 months of follow-up. A composite endpoint involving the Schirmer-I test, unstimulated whole salivary flow, fatigue, erythrocyte sedimentation rate (ESR), and immunoglobulin G (IgG) was devised to provide a clinically meaningful assessment of response, defined as a ≥20% improvement in at least two of the aforementioned parameters, with ≥20% reduction in ESR and/or IgG considered as a single combined criterion. Fourteen patients received all infusions without significant reactions, 1 patient received 3, and another was discontinued due to a mild acute reaction after receiving a partial infusion. Three patients showed moderately elevated levels of Human anti-human (epratuzumab) antibody not associated with clinical manifestations. B-cell levels had mean reductions of 54% and 39% at 6 and 18 weeks, respectively, but T-cell levels, immunoglobulins, and routine safety laboratory tests did not change significantly. Fifty-three percent achieved a clinical response (at ≥20% improvement level) at 6 weeks, with 53%, 47%, and 67% responding at 10, 18, and 32 weeks, respectively. Approximately 40%–50% responded at the ≥30% level, while 10%–45% responded at the ≥50% level for 10–32 weeks. Additionally, statistically significant improvements were observed in fatigue, and patient and physician global assessments. Further, we determined that pSS patients have a CD22 over-expression in their peripheral B cells, which was downregulated by epratuzumab for at least 12 weeks after the therapy. Thus, epratuzumab appears to be a promising therapy in active pSS, suggesting that further studies be conducted.

## Introduction

Primary Sjögren's syndrome (pSS) is a systemic autoimmune disease with a population prevalence of approximately 0.5% [1]. The lymphoid infiltrates within the inflamed tissues contain ectopic germinal center-like structures in 20% of patients [2]. These structures consist of T- and B-cell aggregates containing proliferating lymphocytes, follicular, dendritic, and activated endothelial cells [3]. B-cell homeostasis is disturbed in pSS, with diminished frequencies and absolute numbers of peripheral CD27^+ ^memory B cells. However, the infiltrating B cells are mainly CD27^+ ^memory B cells and CD27^high ^plasma cells [4-6]. This altered B-cell subtype recirculation from inflamed tissue was confirmed recently by Hansen *et al*. [7]. Thus, although pSS is considered to be a T-cell-mediated disease, high levels of B-cell autoreactivity have been associated with high disease activity, the development of systemic complications, and an increased risk of development of B-cell lymphoma [8].

This has led to anti-B-cell monoclonal antibody immunotherapy emerging as a promising new treatment modality in pSS and autoimmune disorders [9]. The use of rituximab, a chimeric anti-CD20 antibody, has been reported in small studies and case reports of SS patients with or without associated lymphoma [10-13]. However, serum sickness-like diseases seem to occur in approximately 20% of patients treated with this chimeric antibody [11] and may be of major clinical concern in subjects with a hyperactive immune system.

CD22 is a 135-kDa B-lymphocyte restricted type-I transmembrane sialoglycoprotein of the immunoglobulin (Ig) superfamily, with seven Ig-like domains and three cytoplasmic ITIMs (immunoreceptor tyrosine-based inhibitory motifs) [14]. CD22 appears intracellularly during the late pro-B-cell stage of ontogeny, shifting to the plasma membrane with B-cell maturation. CD22 is expressed at low levels on immature B cells, expressed at higher levels on mature IgM^+^, IgD^+ ^B cells, and absent on differentiated plasma cells. It is strongly expressed in follicular, mantle, and marginal-zone B cells but is weakly present in germinal B cells (reviewed in [15]). The function of CD22 has not been entirely clarified; it acts as a homing receptor for recirculating B cells through the affinity of the lectin-like domains for 2,6-linked sialic acid-bearing glycans and as a B-cell antigen receptor (BCR) down-modulating coreceptor [16,17]. Because dysregulated expression of CD22 could lead to excessive activation of B cells and autoantibody production [17], targeting this coreceptor in systemic autoimmunity appears to be a potentially new therapeutic pathway. Indeed, antagonistic antibodies to CD22 could provoke downregulation of the BCR (by SHP-1 [Src homology phosphatase-1] recruitment) and inhibiting T- and B-cell crosstalk by downregulation of the CD40 pathway [15,18,19].

Epratuzumab (hLL2), a humanised IgG1 monoclonal antibody directed against the CD22 antigen, binds the third cytoplasmic Ig domain of CD22 [20]. Its known mechanism of action is believed to be the downregulation of the BCR, with mechanisms of action differing from rituximab (by CD22 phosphorylation and BCR effects via immobilised Ig crosslinking) [18,21]. Also, epratuzumab depletes circulating B cells when given to patients with non-Hodgkin's lymphoma (NHL) [22] or systemic lupus erythematosus (SLE) [23], but markedly less than rituximab [24], and has been therapeutically active in both diseases.

Based on these considerations, this phase I/II study was undertaken to investigate the safety and potential local and systemic effects of epratuzumab in patients with active pSS. The effect of this treatment on the peripheral B and T cells in this patient population was also assessed.

## Materials and methods

### Study design

This phase I-II, open-label, 18-week study was conducted at two centres. The protocol was approved by the local ethics committees, and all patients enrolled were required to provide signed informed consent. The study included four infusions of epratuzumab (360 mg/m^2^) at 0, 2, 4, and 6 weeks and three follow-up evaluations at 6, 10, and 18 weeks (that is, 1 day, 4 weeks, and 12 weeks after the fourth infusion). Additionally, a final long-term evaluation was scheduled at 32 weeks (6 months post-treatment).

### Patient population

Males or non-pregnant, non-lactating females, at least 18 years of age, were eligible to participate provided that they fulfilled the American/European consensus group classification criteria for pSS [25]. For females of childbearing potential, a negative pregnancy test result and adequate contraception during the study and for 6 months after the last infusion were required. Additionally, all patients had to demonstrate active pSS prior to study entry. Because there are no validated disease activity criteria for pSS, this was defined as increased B-cell activity, IgG greater than 1.4 g/l or erythrocyte sedimentation rate (ESR) greater than 25 mm/hour, in combination with the presence of autoantibodies. In addition, the patients must have been on symptomatic treatment for at least 6 months prior to screening. Disease-modifying drugs, such as hydroxychloroquine, methotrexate, cyclosporin, sulfasalazine, or corticosteroids, were not allowed during the study and were discontinued at least 4 weeks before study entry. No prior treatment with rituximab or other anti-B-cell antibodies was allowed. The exclusion criteria included serious infections in the previous 3 months, documented HIV or hepatitis-B or -C infection, known malignancy, severe or uncontrolled concurrent disease, and the presence of any other autoimmune/connective tissue disease.

### Study drug administration

Epratuzumab at the dose of 360 mg/m^2 ^in 250 ml 0.9% sterile NaCl was prepared by the hospital pharmacy (Erasme University Hospital and Charite Humboldt University Hospital). The total dose was to be given throughout a 40-minute period. To minimise hypersensitivity, patients were premedicated with acetaminophen (0.5–1 g) and antihistamine (25–50 mg *per os *or intravenous polaramin). Four intravenous infusions of epratuzumab were given at 0, 2, 4, and 6 weeks.

### Concomitant medications

Patients were allowed to continue artificial tears (ATs) and artificial saliva substitutes or nonsteroidal anti-inflammatory drugs provided that the dosage and schedule regimens were stable for at least 4 weeks and were monitored during the study.

### Clinical assessment

Clinical, ophthalmological, and biological evaluations were performed at study entry and at 6, 10, and 18 weeks (that is, 1 day, 4 weeks, and 12 weeks post-treatment). A final evaluation at 32 weeks was also scheduled for patients who were still in long-term follow-up. Clinical assessment (performed by the same physician) consisted of a general physical examination; a dry mouth evaluation (0–2 scale: 0 = none, 1 = mild to moderate, and 2 = severe) involving the collection of unstimulated whole saliva throughout a 15-minute interval by using the spitting technique according to established methods [26] (note: saliva samples were weighed on an analytical balance to determine the volume of saliva obtained, using the conversion formula 1 g = 1 ml); a dry eye evaluation (0–2 scale: 0 = no symptoms, 1 = mild to moderate symptoms relieved by ATs, and 2 = severe symptoms unrelieved by AT); the Schirmer-I test; evaluation of fatigue by a 0–100 mm visual analogue scale(VAS) and a questionnaire (0 = no fatigue, 1 = mild fatigue not interfering with daily activities, 2 = moderate fatigue that interferes with daily activities, and 3 = fatigue with severely reduced activities); the tender/swollen joint count (maximum 36); and the tender point count (maximum 18). The patient's pain assessment and the patient's and physician's global assessments were evaluated by a 0–100 mm VAS. The following biological parameters were measured throughout the study: ESR, C-reactive protein (CRP), complete blood count, renal and liver function tests, creatine phosphokinase, serum Igs (A, M, and G), antinuclear antibodies (ANAs), rheumatoid factor (RF), and peripheral blood B- and T-cell counts.

For purposes of efficacy evaluation, we focused on the main parameters that consisted of the the following parameters: Schirmer-I test, unstimulated whole salivary flow (USF), fatigue VAS, and the laboratory parameters (ESR and IgG). To assess the overall efficacy of epratuzumab in the treatment of pSS, a composite endpoint involving all five parameters was devised to provide a clinically meaningful definition of response. Specifically, a patient was deemed to be a responder if he/she experienced improvement of 20% or more in at least two of the aforementioned parameters, with reduction of at least 20% in ESR and/or IgG contributing jointly as a single combined criterion. Additional assessments of the efficacy data were also performed using improvements of at least 30% and at least 50% in the efficacy parameters.

### Determination of B-cell populations by flow cytometry

Monoclonal antibodies used in this study were CD19 allophycocyanin (APC) (clone SJ25C1) and CD22 phycoerythrin (PE) (clone S-HCL-1). The median channel of fluorescence on the 256-channel linear scale was employed to define numerically the fluorescence distribution of CD22 in a semi-quantitative approach. We routinely used a four-color FACScalibur with automatic loader, driven by Cellquest software that was set up using the three-color FACScomp software and Calibrite microbeads (Becton, Dickinson and Company, San Jose, CA, USA). The stability of the fluorescence intensity signal over a long period of time was assessed using Quantum 1000 microbeads (weekly) and daily with the Rainbow calibration beads from Spherotec (Libertyville, IL, USA) without changing the PMT (photomultiplier tube) voltage and compensations. At the beginning of each new lot of beads, we determined an acceptable range by running aliquots of all beads 10 times and calculating the mean ± standard deviation (SD) and the CV (coefficient of variation) for that lot of beads. For each lot, we determined a mean target channel value for monitoring of flow cytometer performance. Between lots, flow cytometer settings were adjusted to restart the monitoring with the same target channel value as before. In a previous study with 35 hematological patients sampled more than three times during their clinical course (with a mean inter-visit interval of 123 days and range of 13–638 days) and a mean duration of the survey of 492 days (range 13–1,022 days), we observed an inter-contact variation of only 6.2% for CD5 (which has a sharp distribution in CLL [chronic lymphocytic leukemia]) and 6.1% for CD20. Considering the low scattering of CD22 expression on normal B-lymphocytes, we determined a range of normal values and the median CD22 fluorescence on 33 blood samples from normal healthy volunteers. Because the distribution of the median intensity is normal, we determined ± 2 SD of the distribution to define the normal median fluorescence values from a normal range between channels 159 and 178.

Tri-color immunophenotyping of B-lymphocytes was performed with predetermined combinations of murine monoclonal or rabbit polyclonal (for the Ig light chain staining) antibodies directly conjugated with fluorescein isothiocyanate, PE, and CD19 APC as a marker for B-lymphocytes. A lysed and washed whole-blood technique was used. In this procedure, 50 μl blood samples were incubated with the Mab combination at room temperature for 15 minutes. The red blood cells were then lysed using 500 μl ammonium chloride lysing solution. Cells gated in the mononuclear area in a forward-versus side-scatter dot-plot and also those present in a region around the CD19-positive side-scatter low events were considered to be B cells.

### Safety assessments

During the infusion and for 1 hour afterward, the patients were monitored for adverse reactions and vital signs (blood pressure, pulse, and temperature) every 30 minutes. At each visit, patients were asked about any adverse events (AEs) that they experienced. Analysis of pharmacokinetics consisted of epratuzumab levels measured at 30 minutes prior to and after each infusion and at 6, 8, 9, 10, 14, and 18 weeks. Human anti-human (epratuzumab) antibodies (HAHAs) were assessed at study entry and at 6, 10, and 18 weeks.

### Determination of anti-Ro and anti-La autoantibodies

An indirect immunofluorescence procedure using HEp-2000 cells was employed to detect the presence and titer of ANA (Immunoconcept, Sacramento, CA, USA). Anti-Ro/SS-A and anti-La/SS-B antibodies were detected both by fluroro-enzymo-immuno assay (Phadia AB, Uppsala, Sweden) and homemade double immunodiffusion. The serum levels of RF were evaluated by laser nephelometry (N Latex RF; Dade Behring, Inc., Deerfield, IL, USA). Those evaluations were performed at study entry and at 6, 10, and 18 weeks (that is, 1 day, 4 weeks, and 12 weeks post-treatment).

### HAHA assay

The sponsor's [Immunomedics, Inc., Morris Plains, New Jersey, USA] HAHA test is a competitive enzyme-linked immunosorbent assay (ELISA) in which the capture reagent is epratuzumab and the probe is an anti-epratuzumab-idiotype antibody. The anti-idiotype antibody is an acceptable surrogate for what is reacted against in an immunogenic response by humans against the binding portion of epratuzumab which distinguishes the molecule from other human antibodies (that is, the framework region that has human amino acid sequences). Test results are derived from an eight-point standard curve with varying dilutions of anti-idiotype antibody in bovine serum albumin. Patient serum samples are diluted 1:2 with bovine serum albumin and assayed in triplicate. The anti-idiotype standard curve is used to determine the presence of HAHA in unknown samples. An acceptable assay is based on linear regression parameters that must be met to define a valid assay.

### Statistical analysis

In general, discrete variables, including responder rates and AEs, were summarised using frequency counts and percentages. Percentage changes in individual efficacy parameters, B- and T-cell counts, Igs, duration of infusion times, and other continuous numerical variables were summarised using descriptive statistics. The Wilcoxon signed rank test was used to assess the statistical significance of changes in the subjective efficacy measures (VAS scores), tender points, tender joints, ESR, CRP, B cells, T cells, and Igs, compared with their baseline values. All statistical tests used a significance level of ≤0.05.

## Results

### Demographics and baseline disease characteristics

Sixteen patients who met the inclusion/exclusion criteria were to receive four infusions of 360 mg/m^2 ^epratuzumab once every 2 weeks. One patient was discontinued from the study upon receiving a partial dose of the first infusion due to a moderate acute reaction (considered as serious due to a mandatory brief overnight hospitalisation for observation). The ensuing discussion, except for safety, will focus on 15 patients who received at least one infusion of the study drug and had at least one follow-up evaluation. Their baseline characteristics are summarised in Table 1. Among the more notable medical histories, seven patients had pulmonary involvement, five had parotid enlargement, five had thyroid disease, four had neurological impairment, two had Raynaud's disease, and one had type-II cryoglobulinemia.

### Efficacy

Fourteen patients completed the study through the 18-week (12 weeks post-treatment) evaluation period, although one among these missed the 10-week (4 weeks post-treatment) visit. The remaining patient did not receive the fourth infusion but subsequently returned for evaluation at 6 weeks. A total of 10 patients returned for the final evaluation at 32 weeks (6 months post-treatment).

#### General response

**Figure 1:**
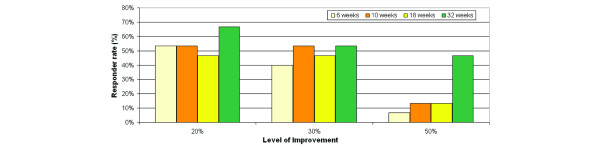
Responder rates. The overall response of a patient was determined using the four domains: dryness of the eyes (Shirmer-I test), dryness of the mouth (unstimulated whole salivary flow), fatigue (visual analogue scale), and laboratory (erythrocyte sedimentation rate and/or immunoglobulin G). A patient who achieved at least 20% improvement in at least two domains is considered a responder.

To assess the overall efficacy of epratuzumab in the treatment of pSS, a composite endpoint involving the Schirmer-I test, USF, fatigue VAS, and the laboratory parameters (ESR and IgG) was devised to provide a clinically meaningful definition of response, as defined in Materials and methods. Of all patients who received at least one dose of the study drug (*n *= 15) (Figure 1), more than half (53%) experienced a clinically meaningful response of improvement of 20% or more in two out of the four criteria at the first post-treatment evaluation at 6 weeks (24 hours after the fourth infusion). The same level of response was maintained through 10 weeks and decreased only slightly at 18 weeks (47%). At the final 32-week evaluation, 67% of the patients still showed a response that satisfied the response criteria. The corresponding results, when the response rates are calculated based on only available patients, are 53%, 62%, 50%, and 91% at 6, 10, 18, and 32 weeks, respectively.

The corresponding response rates based on improvements of at least 30% and 50% also are shown in Figure 1. Remarkably, approximately 50% of the patients also achieved the stricter responder criteria of improvement of at least 30% in two of four efficacy parameters by 10 weeks and continued to do so through 32 weeks. The corresponding response rates based on improvement levels of at least 50% were 10%–45%, depending on the visit.

#### Functional assessments

##### Schirmer-I test

**Figure 2:**
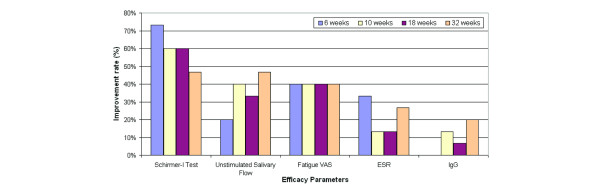
Improvement rates in individual efficacy parameters. The improvement rate is based on achieving an improvement of at least 20% from baseline. ESR, erythrocyte sedimentation rate; IgG, immunoglobulin G; VAS, visual analogue scale.

More than half the patients (Figure 2) improved by at least 20% on their lacrimal function through 18 weeks, with 7/15 (47%) still showing improvement at the final visit at 32 weeks. The corresponding improvement rates based on available patients are 73%, 69%, 64%, and 64% at 6, 10, 18, and 32 weeks, respectively.

##### USF

Improvement by at least 20% in USF was observed in 20%–40% of the patients through 18 weeks (Figure 2), with 7/15 (47%) still showing improvement at the final visit at 32 weeks. The corresponding improvement rates based on available patients are 20%, 46%, 36%, and 64% at 6, 10, 18, and 32 weeks, respectively.

#### Subjective assessments

##### Fatigue and patient and physician global assessments

Improvement by at least 20% in fatigue was consistently observed in 40% of the patients through 32 weeks (Figure 2). The corresponding improvement rates based on available patients are 40%, 46%, 43%, and 55% at 6, 10, 18, and 32 weeks, respectively. Complete results on all subjective efficacy measures as assessed by VAS are summarised in Table 2. Statistically significant improvement from baseline was observed in fatigue and in patient and physician global assessments at several time points. There were no notable changes in pain.

#### Objective assessments of joint counts

Complete results on objective efficacy measures as assessed by joint counts are summarised in Table 3. In general, there were no notable changes except for a statistically significant improvement from baseline in the number of tender joints at 32 weeks.

#### Laboratory assessments

##### ESR

Improvement by at least 20% in ESR was observed in 13%–33% of the patients through 18 weeks (Figure 2), with 4/15 (27%) still showing improvement at the final visit of 32 weeks. The corresponding improvement rates based on available patients are 33%, 15%, 14%, and 36% at 6, 10, 18, and 32 weeks, respectively.

##### CRP

There were no statistically significant changes in CRP at any visit (Table 4).

##### IgG

No improvement in IgG was observed at 6 weeks, but improvement was seen subsequently in 13%, 7%, and 20% of the patients at 10, 18, and 32 weeks, respectively (Figure 2). The corresponding improvement rates based on available patients are 0%, 15%, 7%, and 27% at 6, 10, 18, and 32 weeks, respectively.

#### Lymphocytes and Igs

**Figure 3:**
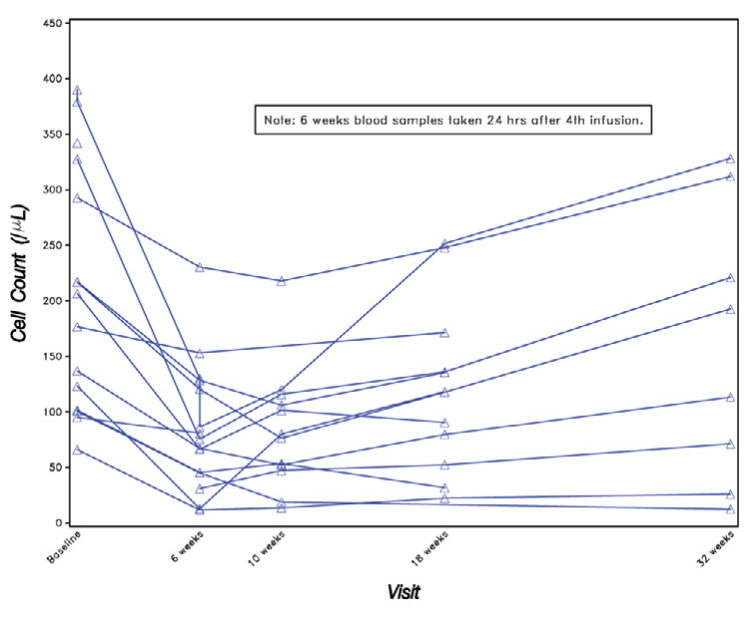
Peripheral B-cell counts.

At study entry, peripheral blood lymphocyte and serum Ig levels (mean ± SD) for the 15 patients were as follows: 211 ± 111 B cells per μl, 1034 ± 426 T cells per μl, 1909 ± 669 IgG mg/dl, 291 ± 111 IgA mg/dl, and 146 ± 54 IgM mg/dl. As shown in Table 5 and Figure 3, mean B-cell levels decreased by 54% at 6 weeks, which persisted at subsequent evaluations, with no evidence of onset of recovery by the final evaluation at 32 weeks (6 months post-treatment). In contrast, there were no consistent patterns of decreases/increases either in the T-cell levels or in the available serum levels of IgG, IgA, and IgM after treatment.

**Figure 4:**
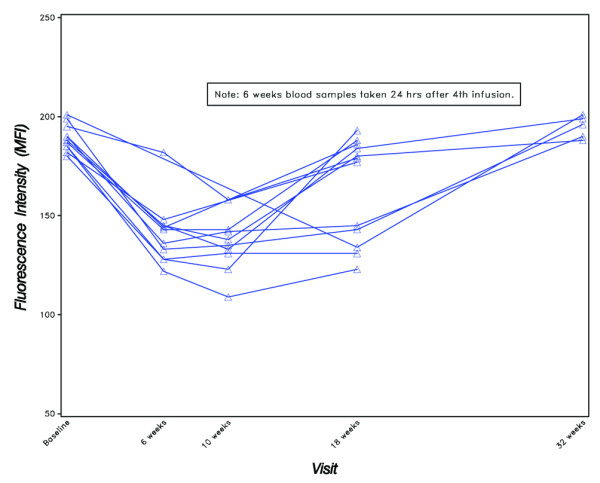
CD22 expression on B cells as measured by mean fluorescence intensity (MFI).

At study entry, all of the patients with available measurements had a CD22 median fluorescence intensity above the normal range (from median 88 to 201). This is consistent with the finding that patients with Sjögren's syndrome have an over-expression of CD22. Twenty-four hours after treatment with epratuzumab at 6 weeks, all but one patient exhibited a decreased CD22 fluorescence intensity below the normal range (Figure 4). At week 18, five patients remained CD22 downregulated, but the others returned to a fluorescence intensity as high as at entry. At the final evaluation, all the patients recovered to the same increased CD22 expression compared with normal, as observed in patients with untreated pSS.

#### Autoantibodies

There were no changes in the autoantibodies, anti-Ro and anti-La, in any patients with at least one post-treatment measurement; specifically, in subsequent visits, no patient developed new autoantibodies that were not also detected at study entry. Almost all patients (13/15 with at least one post-treatment measurement) had measurable ANA titers (1:80 to 1:10,000) at study entry. At subsequent evaluations, including the 32-week (6 months post-treatment) visit, eight patients exhibited at least twice their baseline ANA titers at one or more evaluations, whereas a similar number (seven patients) had not more than half their baseline titers at one or more evaluations, with two of the aforementioned patients having both increases and decreases from their baseline titers at different evaluation time points.

### Study drug administration and reactions

A total of 16 patients were exposed to the study drug. One patient experienced a moderate-severe acute reaction (flushing, dyspnea, nausea, vomiting, nasal mucosa swelling, and glottis pressure) during the first infusion and was discontinued from the study. Of the remaining 15 patients, 14 (93%) completed all four infusions of 360 mg/m^2 ^of epratuzumab and one prematurely terminated the third infusion after experiencing a moderate grade-3 acute infusion reaction (with a loss of consciousness for several seconds) that subsided within 1 hour (the fourth infusion was not administered to this patient). Overall, the infusions were administered in a median infusion time of 45 minutes (20–150 minutes) and were generally well-tolerated, with four transient AEs (headache, lower limb paresthesia, and two cases of acute infusion reaction) that resolved quickly.

### Safety

Safety assessments focus on all 16 patients who were exposed to study medication.

#### Adverse events

During or after treatment, a total of 10 patients reported AEs. Four reported having a serious AE (drug-related: acute infusion reaction as noted above; non-drug-related: dental abscess, transient ischemic attack with secondary seizure, and osteoporotic fracture), and three patients had a non-serious AE considered drug-related (headache, paresthesia, and acute infusion reaction as noted above) that resolved quickly. The remaining AEs considered unrelated to study medication included fever, palpitation, bone pain, sinusitis, carpal tunnel syndrome, diarrhea, and dyspepsia. The two cases of infection reported above (sinusitis and dental abscess treated with intravenous antibiotics) resolved subsequently without any sequelae.

#### Safety laboratories

Standard safety laboratories showed no consistent pattern of change from baseline, and infrequent post-treatment increases in National Cancer Institute Common Toxicity Criteria (CTC) (version 3.0) toxicity grades for these laboratories were all limited to changes of at most one grade level, except for one patient with lymphopenia that increased from CTC grade 0 to 2.

#### Immunogenicity

HAHA analyses showed three patients with elevated values of HAHA: 116 ng/ml at 32 weeks, 120 ng/ml at 18 weeks, and 130 ng/ml at 18 weeks. These isolated cases of low-level positive HAHA are of uncertain clinical significance because they were not associated with specific clinical signs and symptoms or other apparent toxicities.

### Pharmacokinetics

**Figure 5:**
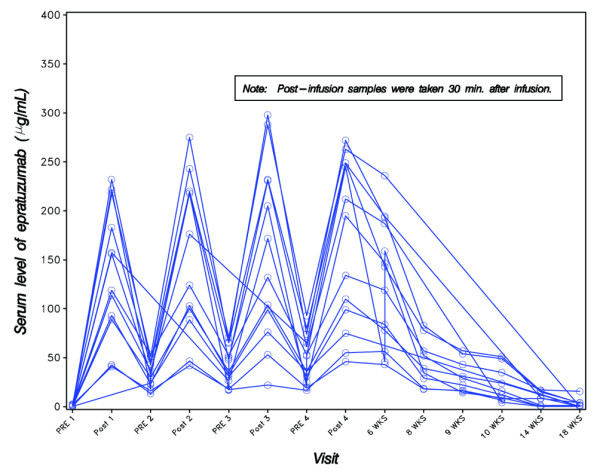
Serum levels of epratuzumab, as measured by enzyme-linked immunosorbent assay.

Serum samples for analysis of pharmacokinetics by ELISA were collected pre- and post-infusion as well as at 6 weeks (24 hours after fourth infusion) and 8, 9, 10, 14, and 18 weeks. Epratuzumab serum levels were detectable above the 0.5 μg/ml assay limit in all 13 available samples at 6 weeks, in 10/11 samples evaluated at 10 weeks, in 6/10 samples evaluated at 14 weeks, and in 6/14 samples evaluated at 18 weeks, with median values of 143 μg/ml (range, 43–236) at 6 weeks, 14 μg/ml (4–51) at 10 weeks, 11 μg/ml (1–17) at 14 weeks, and 3.9 μg/ml (1–16) at 18 weeks (Figure 5). Non-compartmental pharmacokinetic analysis indicated a serum half-life (t_1/2_) after the fourth infusion of 15 ± 8 days.

## Discussion

In this phase I/II open-label study, selective immunomodulation of B cells led to improvement of objective and subjective parameters of disease activity in patients with pSS. In the absence of validated disease activity criteria for pSS, we developed a disease activity score based on the most frequent signs and symptoms of the disease. These included four domains: dryness of the eyes (Schirmer-I test), dryness of the mouth (USF), fatigue (VAS), and laboratory parameters of ESR and/or IgG. Based on this activity score, we observed that more than half (53%) of the patients achieved at least a 20% improvement in at least two domains 24 hours after the fourth infusion at 6 weeks, with the corresponding response rates of 53%, 47%, and 67% at 10, 18, and 32 weeks, respectively. Most improvements occurred in the Schirmer-I test, USF, and fatigue VAS. There appear to be only a few significant changes in ESR or IgG that impacted on the efficacy outcomes. Approximately 40%–50% responded at the improvement level of at least 30%, whereas 10%–45% responded at the 50% improvement level for 10–32 weeks. Interestingly, the number of responders (at 20%, 30%, or 50% improvement levels) was higher 6 months after the treatment administration than earlier. Such results might indicate recovery or regeneration of glandular tissue, suggesting the need for pre- and post-therapy biopsies of minor salivary glands. Additional findings in terms of statistically significant improvement from baseline in fatigue, and in patient and physician global assessments at several time points, also serve to reinforce the positive results based on the composite efficacy endpoint.

B-cell targeting therapies are promising in the treatment of autoimmune diseases, including rheumatoid arthritis [27] and SLE [23]. Also, two studies and some case reports evaluated the anti-CD20 monoclonal antibody, rituximab, in pSS [10-13]. Pijpe *et al*. reported observing a significant improvement in subjective and objective measures, including subjective reports of dryness, fatigue, and salivary flow, mainly in patients with early-onset disease and in only a few with pSS-associated MALT (mucosa-associated lymphoid tissue) lymphoma [11]. Immunologic analysis showed rapid decrease in peripheral B cells but no change in IgG levels. However, four of 14 patients developed human anti-chimeric antibodies (HACAs) and three of them developed a clinical picture compatible with serum sickness [11]. In a retrospective study of short-term efficacy of rituximab in autoimmune diseases, serum-sickness-like diseases occurred in a patient with pSS and in two patients with SLE [12]. This is supported by another French, open-label study using low-dose rituximab which reported improvement in subjective parameters of dryness and which also showed that two of 16 patients developed a clinical picture of serum sickness [13]. The most striking finding in those studies is the observation of HACA-associated serum sickness, which may be of major clinical concern in future trials. Accordingly, fully humanised anti-CD20 monoclonal antibodies are under evaluation in autoimmune diseases, in addition to NHL [28-30]. Nevertheless, all of these CD20 antibodies appear to markedly deplete circulating B cells in treated patients.

Although depleting B cells is interesting in the treatment of autoimmune diseases, a novel and rational approach is modulating their function. Initial data have shown that epratuzumab is effective and safe in the treatment of SLE [23]. This treatment was associated with a modest depletion of B cells (34%–41%) within 18 weeks, as we observed also in the present study (39%–54% within 18 weeks) and as was also found in patients with NHL treated with epratuzumab [22]. However, it might also function by signalling through the inhibitory CD22 molecule, causing down-modulation of BCR signalling, as suggested in recent laboratory studies comparing epratuzumab with rituximab [21]. B-cell homeostasis is disturbed in pSS with diminished frequencies and absolute numbers of peripheral CD27^+ ^memory B cells [4-7]. In addition, we report here, for the first time, that patients with pSS have a CD22 over-expression in their peripheral B cells, which was downregulated by epratuzumab for at least 12 weeks after the therapy.

In addition to assessing any evidence of efficacy, the objective of this open-label phase I/II study was to evaluate the safety of epratuzumab in patients with active pSS. Three patients showed moderately elevated levels of HAHA, but without any specific clinical symptoms or apparent toxicity that could be associated with the elevations. As compared with patients with lymphoma, those suffering from autoimmune diseases have been reported to present a higher rate of antibodies to chimeric rituximab, but usually not related with clinical manifestations [27,31]. These discrepancies may be explained in part by the high B-cell activity in pSS and the lack of concomitant immunosuppressive therapy.

## Conclusion

This initial experience in patients with active pSS demonstrated that four doses of 360 mg/m^2 ^epratuzumab immunotherapy appears to be safe and well-tolerated when infused within 45 minutes, with clinically significant responses observed in approximately half the patients for at least 18 weeks in the presence of modestly decreased (39%–54%) circulating B-cell levels, and with evidence of minimal immunogenicity, as measured by HAHA. We conclude that epratuzumab may be a promising therapy in patients with active pSS and that a multicentre, randomised, double-blinded, controlled study to confirm the beneficial effects of anti-CD22 therapy is indicated.

## Abbreviations

ACR = American College of Rheumatology; AE = adverse event; ANA = antinuclear antibody; APC = allophycocyanin; AT = artificial tear; BCR = B-cell antigen receptor; CRP = C-reactive protein; CTC = [National Cancer Institute] Common Toxicity Criteria; ELISA = enzyme-linked immunosorbent assay; ESR = erythrocyte sedimentation rate; HACA = human anti-chimeric antibody; HAHA = human anti-human (epratuzumab) antibody; Ig = immunoglobulin; NHL = non-Hodgkin's lymphoma; PE = phycoerythrin; pSS = primary Sjögren's syndrome; RF = rheumatoid factor; SD = standard deviation; SLE = systemic lupus erythematosus; USF = unstimulated whole salivary flow; VAS = visual analogue scale.

## Competing interests

SDS and GRB declare research funding for this study provided by Immunomedics, Inc. SDS has acted as a research consultant for Genentech, Inc. NKWT, WAW, and DMG have employment and financial interests (stock) in Immunomedics, Inc., whichowns the antibody tested in this paper. OP and LT declare no competing interests.

## Authors' contributions

All authors contributed to data interpretation and the final manuscript. SDS and GRB were the principal investigators and were responsible for all aspects of the study, including patient selection and performing patient-related study procedures. SDS, GRB, DMG, and WAW designed the clinical trial protocol, and NKWT was responsible for data management and statistical analysis. All authors read and approved the final manuscript.

## Figures and Tables

**Table 1:** Patient demographics and baseline disease characteristics

**Parameters**	***n *= 15**
Gender (female/male)	13/2
Age (years)	49 (33–73)
Median years post-diagnosis	2.9 (1–16)
Ocular symptoms	15 (100%)
Schirmer-I test (mm)	12 ± 12
Oral symptoms	15 (100%)
Unstimulated salivary flow (ml/minute)	0.07 ± 0.13
Moderate-to-severe fatigue	13 (87%)
Fatigue VAS (mm)	56 ± 22
Focus score ≥ 1	12 (80%)
Anti-Ro antibodies	12 (80%)
Anti-La antibodies	11 (73%)
ESR (mm/hour)	33 ± 15
IgG (mg/dl)	2,114 ± 934

ESR, erythrocyte sedimentation rate; Ig, immunoglobulin; VAS, visual analogue scale.

**Table 2:** Subjective measures assessed by visual analogue scale (0–100 mm)

**Parameters**	**Baseline (*n *= 16)**	**6 weeks (*n *= 15)**	**10 weeks (*n *= 13)**	**18 weeks (*n *= 14)**	**32 weeks (*n *= 11)**
Fatigue	55 ± 21	46 ± 30	44 ± 28	45^a ^± 22	41^a ^± 29
Pain	49 ± 28	31 ± 32	32 ± 29	39 ± 25	34 ± 26
Patient assessment^b^	62 ± 29	38^a ^± 30	49 ± 23	48^a ^± 31	40^a ^± 28
Physician assessment^c^	56 ± 16	30^a ^± 22	36^a ^± 14	31^a ^± 20	26^a ^± 12

Results are given in mean ± standard deviation. ^a^Denotes statistical significance of the observed median change-from-baseline value with *P *≤ 0.05 by Wilcoxon signed rank test. ^b^Patient self-assessment of overall well-being. ^c^Physician global assessment of patient's overall well-being.

**Table 3:** Number of tender points and tender joints

**Parameters**	**Baseline (*n *= 16)**	**6 weeks (*n *= 15)**	**10 weeks (*n *= 13)**	**18 weeks (*n *= 14)**	**32 weeks (*n *= 11)**
Tender points	4.1 ± 5.6	2.0 ± 3.1	2.1 ± 3.4	1.6 ± 2.9	2.4 ± 3.3
Tender joints	4.0 ± 7.5	1.0 ± 1.8	1.1 ± 1.6	1.2 ± 1.9	0.3^a ^± 0.5

Results are given as mean ± standard deviation. ^a^Denotes statistical significance of the observed median change-from-baseline value with *P *≤ 0.05 by Wilcoxon signed rank test. Almost all patients had no swollen joints at baseline or subsequent time points.

**Table 4:** Post-treatment changes in CRP, ESR, and Igs

	**Post-treatment change from baseline (mean ± standard deviation)**			
	
**Parameters**	**6 weeks**	**10 weeks**	**18 weeks**	**32 weeks**
	***n *= 13**	***n *= 13**	***n *= 12**	***n *= 10**
CRP	0.42 ± 1.39	-0.02 ± 0.33	0.19 ± 0.60	0.03 ± 0.22
ESR	1.3 ± 15.0	1.5 ± 11.0	-0.9 ± 7.7	-0.3 ± 11.6
				
	***n *= 15**	***n *= 13**	***n *= 13**	***n *= 9**
IgG	29.7 ± 249.6	-31 ± 379.6	7.1 ± 313.5	-120.8 ± 363.3
IgA	1.1 ± 24.4	7.8 ± 46.9	-1.2 ± 59	-3.8 ± 62.4
IgM	-14.9 ± 25.3	-9.6 ± 28.3	-15.0 ± 32.8	-2.3 ± 34.8

None of the changes from baseline in the above parameters was statistically significant. CRP, C-reactive protein; ESR, erythrocyte sedimentation rate; Ig, immunoglobulin.

**Table 5:** Post-treatment changes in lymphocytes

	**Post-treatment percentage change from baseline (mean ± SD)**			
	
**Parameter**	**6 weeks**	**10 weeks**	**18 weeks**	**32 weeks**
Lymphocytes	***n *= 14**	***n *= 12**	***n *= 11**	***n *= 7**
B cells	-54%^a ^± 25%	-45%^a ^± 47%	-39%^a ^± 23%	-31%^a ^± 33%
T cells	2% ± 36%	-6% ± 34%	1% ± 13%	-5% ± 19%

^a^Denotes statistical significance of the observed median change-from-baseline value with *P *≤ 0.05 by Wilcoxon signed rank test. None of the changes from baseline in T cells was statistically significant. SD, standard deviation.
